# *Saline Systems*: A research journal bridging gene systems and ecosystems

**DOI:** 10.1186/1746-1448-1-1

**Published:** 2005-07-04

**Authors:** Shiladitya DasSarma

**Affiliations:** 1 University of Maryland Biotechnology Institute, Center of Marine Biotechnology, Baltimore, Maryland 21202 USA

## Abstract

*Saline Systems *addresses the publication needs of scientists conducting basic and applied research on coastal and inland saline environments and their flora and fauna. The journal covers research at all levels, from individual genes to whole genomes and entire ecosystems. Rapid progress in the molecular biology and microbial ecology of halotolerant and halophilic organisms and the sensitivity of many saline environments warrants an online journal with fast turnaround times. Many saline environments are threatened and the need for an Open Access journal to address the dissemination and sharing of knowledge on their conservation and management is compelling. *Saline Systems *provides an interdisciplinary forum for scientists working within all of the relevant fields.

## 

*Saline Systems *is being launched to address the publication needs of researchers exploring all aspects of saline environments. The journal is timely because rapid progress is being made in the molecular biology, genomics, and ecology of halotolerant and halophilic organisms inhabiting saline environments [1,2]. The advent of high-throughput technologies, such as genome sequencing, DNA microarrays, and proteomics, is beginning to revolutionize our understanding of these complex systems. Genomes of many halophilic species have already been completely sequenced and several have been subjected to detailed bioinformatic, reverse genetic, and transcriptome analyses. Metagenomic and metaproteomic studies are also yielding insights into the rich metabolic and ecological diversity of saline ecosystems.

Saline environments, including coastal and inland ecosystems, harbour a substantial fraction of the biodiversity of the world. These serve as a major source of food for humankind through commercial fishing and aquaculture. About 3 billion people live near coastal communities, and escalating population growth and development are degrading the habitats critical to biodiversity and threatening the sustained vitality of coastal economies [3]. Estuarine and coastal wetlands are disappearing at an alarming rate and arable lands are increasingly stressed by high salinity [4]. Greater understanding of these environments is a prerequisite for protecting both biodiversity and economic vitality. In order to address these concerns, better communication is required between scientists working at all different levels.

## *Saline Systems*: Integration of saline environments and systems biology

The primary aim of the journal is to enhance communication between and among scientists concerned with saline environments. Integration is intended to foster a deeper understanding of these critical environments world-wide, and of the halophilic and halotolerant organisms which inhabit them, as well as the underlying fundamental processes operating in them. Integration of knowledge at various levels is the hallmark of the systems biology approach. Such an approach permits information from interdisciplinary studies, from genetics and genomics, through physiology and biochemistry, to ecology and environmental biology, to be considered together, and sets the stage for predictive modelling of systems.

The environments of concern to *Saline Systems *include both coastal and inland regions, including natural lakes, marshes, springs, lagoons, and estuaries, and solar panes and other evaporitic and arid environments, and also their diverse micro- and macro-flora and -fauna. Specific approaches and research areas of interest include (1) the genomics, molecular biology, and environmental biology of halophilic and halotolerant organisms; (2) the limnology of salt lakes including microbial ecology, biogeochemical cycling, paleolimnology, and trophic and ecosystem dynamics; (3) the biodiversity, conservation, and resource management of saline environments; and (4) biotechnological applications of saline environments, including aquaculture.

The expertise of the editorial board of *Saline Systems *covers the entire scope of the journal. The editorial board membership includes experts in the chemistry and biology of saline environments, including general limnology and biogeography, nutrient cycling, ecological modelling, and biostatistics, biology, taxonomy and phylogeny of halophilic archaea and bacteria, biology and ecology of halotolerant eukaryotes, including algae, fungi, protists, invertebrates, and plants, biology of *Artemia*, crustaceans, fish, and waterbirds, genomics and postgenomics of micro- and macroflora and fauna of saline environments, and aquatic conservation biology.

## *Saline Systems*' policy of Open Access

*Saline Systems *is an Open Access, online journal publishing high quality manuscripts on all aspects of basic and applied research on halophilic organisms and saline environments. A wide range of article types are published in the journal: research, book reviews, database articles, commentaries, methodology articles, short reports and reviews. Articles in *Saline Systems *are submitted and peer-reviewed via an online manuscript handling system, and accepted articles are published online immediately upon acceptance. In addition to the journal web site, the full text of each Open Access article is permanently archived in online repositories separate from the journal, including PubMed Central [5], the US National Library of Medicine's repository of life science literature. Thus, all articles published in *Saline Systems *become universally accessible online, and so an author's work can be freely read by anyone at no cost. Moreover, the authors hold copyright for their work, and they may grant anyone the right to reproduce and disseminate the article, provided that it is correctly cited and no errors are introduced [6].

*Saline Systems*' policy of Open Access is highly beneficial for science and the general public. Traditional barriers to accessing research results are removed, resulting in dissemination to the widest possible audience. Authors become free to reproduce and distribute their work, for example on their institution's website. Articles are accessible beyond the traditional scientific audiences through increasingly powerful web search engines, which likely results in greater citations and higher impact [7,8]. Access to articles published in *Saline Systems *is not limited by library budgets, which are increasingly strained. Research published in the journal is accessible to all citizens, not just those with access to a library with a subscription. Importantly, scientists from resource-poor countries and institutions, as long as they have a connection to the internet [9], can access articles published in *Saline Systems*. It is very likely that our policy of Open Access will contribute toward levelling the scientific playing field and advance science.

Another important policy of *Saline Systems *is that decisions about a manuscript are based solely on the quality of the work, not on whether the authors' can pay the article-processing charge. A fraction of the papers published in *Saline Systems *will have publication fees waived, at the discretion of the editors. Editors have no financial incentive or competing interest in articles published in the journal, and journal policies are intended primarily to advance science. We invite the entire community of scientists to contribute to the success of this timely field and its Open Access journal, *Saline Systems*.
